# Understanding the Lived Experience After Colectomy and Ileal Pouch-Anal Anastomosis for Ulcerative Colitis: A Qualitative Study

**DOI:** 10.1093/crocol/otaf007

**Published:** 2025-01-25

**Authors:** Edward L Barnes, Marcella H Boynton, Darren A DeWalt, Erica Brenner, Hans H Herfarth, Michael D Kappelman

**Affiliations:** Division of Gastroenterology and Hepatology, University of North Carolina at Chapel Hill, Chapel Hill, NC, USA; Multidisciplinary Center for Inflammatory Bowel Diseases, University of North Carolina at Chapel Hill, Chapel Hill, NC, USA; Center for Gastrointestinal Biology and Disease, University of North Carolina at Chapel Hill, Chapel Hill, NC, USA; Division of General Medicine and Clinical Epidemiology, University of North Carolina at Chapel Hill, Chapel Hill, NC, USA; Division of General Medicine and Clinical Epidemiology, University of North Carolina at Chapel Hill, Chapel Hill, NC, USA; Multidisciplinary Center for Inflammatory Bowel Diseases, University of North Carolina at Chapel Hill, Chapel Hill, NC, USA; Center for Gastrointestinal Biology and Disease, University of North Carolina at Chapel Hill, Chapel Hill, NC, USA; Division of Pediatric Gastroenterology, University of North Carolina at Chapel Hill, Chapel Hill, NC, USA; Division of Gastroenterology and Hepatology, University of North Carolina at Chapel Hill, Chapel Hill, NC, USA; Multidisciplinary Center for Inflammatory Bowel Diseases, University of North Carolina at Chapel Hill, Chapel Hill, NC, USA; Center for Gastrointestinal Biology and Disease, University of North Carolina at Chapel Hill, Chapel Hill, NC, USA; Multidisciplinary Center for Inflammatory Bowel Diseases, University of North Carolina at Chapel Hill, Chapel Hill, NC, USA; Center for Gastrointestinal Biology and Disease, University of North Carolina at Chapel Hill, Chapel Hill, NC, USA; Division of Pediatric Gastroenterology, University of North Carolina at Chapel Hill, Chapel Hill, NC, USA

**Keywords:** patient reported outcome measures, pouchitis, J-pouch, quality of life

## Abstract

**Background:**

The patient experience after ileal pouch-anal anastomosis (IPAA) for ulcerative colitis (UC) remains poorly defined, resulting in heterogeneity in clinical assessments and research. We performed a qualitative study to better understand the experience of patients after IPAA for UC, with a focus on the symptoms experienced by patients and the resultant effects on quality of life (QoL).

**Methods:**

We conducted semi-structured focus groups among patients who had undergone IPAA for UC. We invited patients with a variety of pouch-related conditions, including patients reporting normal pouch function and those with diagnosed inflammatory conditions of the pouch. We included questions on patients’ experiences and symptoms after IPAA based on 4 thematic areas identified by a previously performed systematic review: bowel symptoms, activities, general issues and quality of life, and psychosocial.

**Results:**

We interviewed 15 individuals over the course of 4 focus groups. Participants described the significant impact that bowel symptoms after IPAA had on other activities including work and daily life, and their subsequent relation to QoL themes. Participants noted symptoms of frequency, urgency, and incontinence after IPAA, and many shared how these symptoms required them to change their lifestyle, particularly by altering their daily schedule or changing their diet. Nevertheless, most participants reported QoL improvement after IPAA.

**Conclusions:**

In this qualitative study evaluating the experience of patients after IPAA, multiple bowel-related symptoms impact other areas of life and overall QoL. Patients undergoing IPAA for UC represent a unique patient population, and thus patient-centered outcome measures should be designed to standardize their assessment.

## Introduction

Proctocolectomy with ileal pouch-anal anastomosis (IPAA) is the most common restorative surgery among patients with ulcerative colitis (UC) who require colectomy for UC-related dysplasia or medically refractory UC. Understanding patient experiences after IPAA is vital to predicting responses to surgery; however, our ability to reliably assess the patient’s lived experience after IPAA has been limited by a reliance on a combination of ad hoc patient-reported outcome (PRO) measures and measures developed for other disease states that have only modest overlap in symptoms.^1^ In prior attempts to quantify the patient experience after IPAA, including among patients with inflammatory conditions of the pouch, researchers have used multiple nonspecific measures leading to inadequate or incomplete assessment and difficulty comparing results across studies or populations. Previously used measures also vary widely in the number and types of constructs being assessed, further complicating our understanding of patient experiences.^1^

A standard measure of overall function after IPAA would allow for a rigorous assessment of patient outcomes across patients, centers, and studies.^2^ In particular, there is a need for assessing the lived experience after IPAA as it relates to inflammatory conditions of the pouch and other pouch-related complications and the subsequent impact on quality of life (QoL).^1^ Much of our understanding of both pouch function and QoL after IPAA surgery relies on assessments in selected populations from single centers repeatedly using the same measures that may have not been validated for patients with an IPAA.^1,3–5^ In recent years, researchers and providers have attempted to better quantify the patient experience and potential bowel dysfunction after IPAA with tools such as the Ileoanal Pouch Syndrome Severity Score.^6^

Whether these measures represent the patient’s experience after IPAA, particularly when experiencing symptoms related to inflammatory conditions of the pouch, is not well understood. The ideal PRO for the assessment of patients with pouchitis and other inflammatory conditions of the pouch would evaluate both pouch symptoms and any subsequent or related impact on QoL. To date, the most widely used PROs among patients after IPAA do not specifically assess pouch symptoms^4,7^ or are not responsive to changes in symptoms after IPAA.^1^

To improve our understanding of the patient experience after IPAA, particularly among patients who experience inflammatory conditions of the pouch, and to inform the development of future PROs for use in research and clinical care, we conducted focus groups with individuals who had undergone a restorative proctocolectomy with IPAA for UC. Our aims were to (1) better understand the patient experience of IPAA, (2) improve our understanding of the most impactful symptoms and issues that patients face in association with inflammatory conditions of the pouch, and (3) evaluate the association between those symptoms and QoL themes.

## Materials and Methods

### Setting and Participants

Eligibility requirements for participation in the focus group sessions included was being between the ages of 18 and 99 years old and having undergone a restorative proctocolectomy with IPAA for UC. Recruitment was conducted in the University of North Carolina (UNC) Multidisciplinary Pouch Clinic and the UNC Multidisciplinary IBD Center. Given our interest in representing the breadth of the patient experience after IPAA, we recruited patients with normal pouch function, intermittent pouchitis, chronic antibiotic-dependent pouchitis, chronic antibiotic-refractory pouchitis, and Crohn’s-like disease of the pouch. Patients were recruited at the time of standard-of-care visits or if they had previously been identified as having a specific pouch-related disorder by their treating provider. We invited patients with confirmed eligibility to participate in either an in-person or virtual focus group session (3 in-person sessions and 1 virtual session were offered) and obtained informed consent. A total of 21 eligible individuals were invited to participate. Three declined due to a lack of interest in participating, three initially agreed to participate but later canceled prior to their respective focus group sessions due to new requirements that would not allow them to participate at the scheduled time, and 15 participated. The goals of the focus group sessions were explained to patients during the consent process and at the time of the interview.

### Data Collection

Between December 2023 and January 2024, four 60-90 minute focus groups were conducted. Each focus group was led by a trained qualitative researcher (ELB, credentials: MD, occupation: gastroenterologist, sex: male) who followed a semi-structured interview guide. The interview guide was developed based on a previously performed systematic literature review identifying the key domains used in PROs evaluating patients after IPAA for UC,^1^ which addressed symptoms experienced among participants after IPAA and the impact of having an IPAA and pouch function on QoL. QoL was evaluated in several previously identified specific domains, including impact on daily activities, fatigue, and psychosocial domains (Table 1). The qualitative researcher had an existing provider-patient relationship with 6 out of 15 (40%) of participants, and his role as interviewer and researcher was explained to all participants during recruitment and at the beginning of each interview session. We audio-record and transcribed both the in-person and virtual interviews. Transcriptions were not returned to participants for comments and/or correction. Pertinent demographic and clinical data were obtained from medical records.

**Table 1.** Example focus group questions, stratified by theme.

Theme	Question
Bowel symptoms	Does your pouch cause you to experience urgency? Do you have frequent bowel movements because of your pouch? Do you ever have bowel accidents because of your pouch? Do you have pain or discomfort from your pouch? Do you have rectal bleeding because of your pouch?
Activities	Does your pouch affect your day-to-day life? Do you ever have to plan your activities around your bowel movements? Does your pouch affect your ability to attend school or work? Do you have to change your plans because of unexpected bowel issues?
General issues and quality of life	How did your bowel function leading up to pouch surgery affect your day-to-day life? Would you say that having pouch surgery has improved your life? Do you have worries about your bowel issues in the future? Are you worried that pouch problems will get worse?
Psychosocial	What kind of feelings about your pouch did you have immediately after pouch surgery? How do you generally feel about your pouch now? Does having a pouch make you feel different from other people? Do you regret having pouch surgery?

### Data Analysis

Following transcription, we analyzed the de-identified interview transcripts using ATLAS.ti qualitative analysis software (ATLAS.ti Scientific Software Development GmbH; Berlin, Germany). Based on the interview guide and prior systematic review, we developed an initial codebook containing thematic areas, concepts, domains, and symptoms. After discussion of planned codes and thematic areas between 2 members of the research team (ELB and MHB), transcripts were reviewed by a single coder (ELB). The coding frame was applied to the text, and the codebook was modified in an iterative process based on information gained through the analysis, with any refining of codes or themes confirmed via discussion amongst two members of the research team (ELB and MHB). We conducted focus groups until we reached thematic saturation.^8,9^ We defined thematic saturation as no new symptoms, themes, or impacts identified in a focus group or on review of transcripts. Given the somewhat subjective nature of thematic saturation, this has previously been quantified using a threshold of ≤5% new information (ie, no new symptoms, themes or impacts) identified in a focus group or on review of transcripts,^9^ thus this threshold was adopted in this study.

Summary memos and analysis notes were collected during the focus groups and during coding to help characterize key concepts identified in the data collection and analysis process. Notes included focus group comments that were beyond the scope of this project but were important to the lived experience after IPAA (eg, the need for psychosocial counseling during the perioperative period, the potential benefit of peer support programs, the impact of IPAA surgery on sexuality and intimacy). The initial transcripts were not reviewed by participants. However, a draft of the results and corresponding manuscript was provided to multiple participants from each focus group so they could provide feedback. This participatory approach led to several improvements, including the identification of areas or topics discussed in the focus groups that were not mentioned in the original version of the manuscript. For example, participant checking led to the inclusion of further information regarding the use of anti-diarrheal medications to aid in controlling symptoms such as bowel frequency.

## Ethical Considerations

All methods and procedures were approved by the University of North Carolina at Chapel Hill Institutional Review Board.

## Results

A total of 15 patients participated in 4 focus groups (3-5 participants per focus group). Baseline demographics and clinical characteristics of participants are depicted in Table 2.

**Table 2.** Demographics and clinical characteristics of participants.

	Focus group participants *n* = 15	
	Median	Interquartile range
Age, in years, at time of focus group	53	43-57
Months since IPAA surgery	40	16-72
	***N***	**%**
*Sex*		
Female	7	47
Male	8	53
*Race*		
Black	1	7
White	14	93
*Pouch-related diagnosis*		
Normal pouch	3	20
Chronic antibiotic-dependent pouchitis	3	20
Chronic antibiotic-refractory pouchitis	4	27
Crohn’s-like disease of the pouch	3	20
Irritable pouch syndrome or motility disorder	2	13
*Focus group interview type*		
In person	12	80
Virtual	3	20

We identified specific themes related to bowel function after IPAA in focus group discussions, which were subsequently tied to QoL themes. These areas of focus were as follows: frequency, incontinence, pain, urgency, QoL, fatigue, sleep, changing plans, daily activities, and effects on school or work. We also analyzed the impact of having an IPAA on psychosocial issues, such as worries, regret, embarrassment, changing feelings about having a pouch over time, and relating to others.

### Bowel Symptoms

#### Frequency

Participants described a number of issues related to bowel frequency after IPAA surgery. Many participants described a normal bowel routine with between 7 and 10 bowel movements per day, noting that an increase of daytime or nocturnal bowel movements correlated with increased inflammation of the pouch. Additionally, although participants noted that their bowel frequency with an IPAA is higher than their peers without IBD or without an IPAA, participants also noted that their bowel frequency had significantly improved since undergoing IPAA:

“*I probably go to the bathroom more than the average person, but my whole quality of life is drastically improved.*”[Focus Group (FG) 1]

“*It’s been fine…frequency, but kind of getting used to it and planning around it.*”[FG2]

It should be noted that multiple participants indicated that their bowel frequency (and sometimes other bowel-related symptoms) were well-controlled only with the addition of medications such as loperamide. This was particularly true if loperamide was used in anticipation of events such as social activities or travel.

#### Incontinence

Incontinence was a major concern for many participants after IPAA surgery, although, it should be noted there was substantial heterogeneity in its severity. Although a few participants had never experienced incontinence, many experienced regular, even daily, incontinence. Nocturnal incontinence was a particular worry for multiple participants because of the impact on sleep and the resultant fatigue. Some participants noted that incontinence symptoms were particularly problematic when stool was loose or when combined with a sensation of urgency.

*There are a lot of people with J-pouches [who] have leakage all the time.* [FG1]

*At night [incontinence] is my problem. I don’t sleep heavy because I am paranoid.* [FG1]

When describing incontinence episodes and experiences, often discussed its emotional impacts, including worry and embarrassment. Participants described concerns regarding the impact of incontinence and the lack of control of this symptom, leading to significant effects on overall QoL:

*Always, you have to worry about accidents.* [FG1]

To combat the effects of incontinence, 3 participants specifically referred to the use of diapers or pads.

*[I have] pouchitis that comes and goes every couple of months. I wear [a diaper] because the back of your pants can get pretty soiled when it’s just running watery. So I do wear a diaper to help.* [FG3]

*I always wear a diaper whether I need it or not because I don’t want to get caught.* [FG3]

*During a flare up and getting in the car, I can still soil, so I have the extra measure of the diaper.* [FG3]

#### Pain

Pain, particularly in the rectal, anal, or perianal areas, remained a common symptom among participants after IPAA surgery. Participants also described new pain that differed from prior discomfort associated with UC.

*I have rectal pain that I never had before the pouch.* [FG1]

Additionally, some participants noted a cramping sensation or discomfort that preceded a bowel movement in addition to a pressure sensation that was more noticeable in the setting of active inflammation.

*I was having a lot of pain, like a lot of cramping when I’d have to go.* [FG2]

Other participants described a discomfort that was difficult to characterize in relation to other pain or sensations they had experienced with UC or other disorders previously:

*I get pain. It’s not consistent. I don’t know how to describe it, but it’s a pain in the lower rectum*. [FG3]

#### Urgency

Participants also expressed a variety of experiences with respect to urgency. Many participants compared their sense of urgency with an IPAA to the prior symptoms of urgency associated with medically-refractory UC. Several participants noted an improved sense of control of bowel movements (and subsequently a decreased sense of urgency) after IPAA surgery as compared to the urgency experienced with medically refractory UC:

*Certainly the godsend was getting rid of the urgency of ulcerative colitis.* [FG2]

However, other participants continued to experience urgency with an IPAA:

*Sometimes if I feel urge to use the bathroom and I’m away and maybe have to wait an hour or two, I’ll still kind of get some pain every now and then.* [FG2]

*I experience urgency, not nearly the scale of what I had with ulcerative colitis, but I do have urgency.* [FG1]

#### Bowel symptoms in participants with inflammatory conditions of the pouch

Participants noted that bowel symptoms were also a major factor in distinguishing an episode of active inflammation related to pouchitis as compared to normal bowel function after IPAA surgery. One participant noted that incontinence was the worst symptom associated with pouchitis.

*I [experienced incontinence] when I was in the worst part of pouchitis.* [FG4]

Other participants reported that increased urgency was a hallmark symptom of pouchitis, with urgency also being a predominant symptom that interrupted sleep.

*It was always at night when… I was waking up to the urgency. And most of the time it wasn’t even that I completely lost control.* [FG4]

When distinguishing normal bowel function from an episode of pouchitis, bowel frequency was identified as an early indicator of pouchitis as well as a problematic symptom among patients with chronic pouchitis.

*I’m starting to recognize the pouchitis is a set of symptoms, you know, increased bowel movement frequency.* [FG1]

*…unless I have a really bad day, which would be really bad diarrhea. My family is so adjusted to me and the way I am.* [FG2]

### Quality of Life

#### Global quality of life

Importantly, most participants noted that although there were issues related to having a pouch or inflammatory conditions of the pouch, their overall QoL tended to improve after IPAA compared to UC or living with an ostomy. Specifically, participants noted that the symptoms of UC prior to colectomy and IPAA were very difficult including frequency, urgency, pain, and their associated effects on QoL and that these improved after IPAA surgery.

*I would say it’s made my life much, much better than it was when I had ulcerative colitis.* [FG1]

*I never have a fantastic day, but the quality of life is so much better than before the pouch.* [FG1]

Some participants also drew connections between improvements in their QoL and to current interventions for the treatment of inflammatory conditions of the pouch, including chronic therapies for chronic pouchitis or Crohn’s-like disease of the pouch, resulting in improvement of bowel frequency or pain:

*If I take the antibiotics that are prescribed, the inflammation pretty much goes away... my health, energy, and all that has gone through the roof.* [FG1]

#### Sleep

Sleep was a major theme that overlapped with several pouch-related symptoms. The impact of incontinence on sleep patterns was among the strongest impacts on QoL noted by participants. This impact on sleep was further influenced by the effects that participants noted of a constant sense of worry about incontinence, even while sleeping.

*It’s embarrassing no matter how old you are to have some sort of leakage or something you can’t control in the middle of the night.* [FG1]

*I sleep on a towel because it worries me that I’m going to soil things.* [FG3]

Additionally, participants who noted increased nocturnal frequency reported worsening issues with sleep disturbance. Participants also noted that issues with sleep caused difficulties with other activities, including work:

*The lack of sleep was affecting my ability to carry out my work activities.* [FG4]

#### Fatigue

Separate from sleep, some participants noted issues with fatigue or a lack of energy after undergoing IPAA surgery. Participants noted that interventions such as iron infusions are commonly used to help with fatigue or a lack of energy, but some participants continued to feel fatigued despite these interventions:

*I’m tired all the time.* [FG1]

*I’m exhausted all the time. I wake up exhausted, and I still fight that a lot.* [FG1]

### Impact on Other Domains

In addition to impacts of IPAA surgery on bowel symptoms and QoL, participants noted several other daily life activities and domains that were affected.

#### Daily activities and changing plans

Many participants relayed the need to plan daily activities around bowel symptoms or pouch function after IPAA. In particular, a few patients noted the need to plan meals around access to a restroom due to post-meal urgency (demonstrating an overlap with the diet theme detailed below).

*I regulate my food intake starting the day before. If I have a special event, like coming [to this focus group session], I stopped eating yesterday about six.* [FG3]

Multiple participants compared these necessary adjustments to daily activities or work functions to accommodate potential bowel dysfunction to those experienced prior to surgery for UC. When specifically discussing the impact on school or work, participants noted an increased ability to participate in meetings post-surgery.

There was variability in describing the impact of IPAA surgery on daily activities. Although some participants shared that they had adopted a routine that did not restrict their activities after IPAA surgery, others reported that they schedule activities around bowel movements. Some participants reported incapacitating symptoms to the extent that they had trouble leaving the house or working after IPAA surgery:

*I just sort of schedule around it so I know I won’t have a bowel movement.* [FG4]

*I pre-plan. Like if my friends want to go on a boat for eight hours, I won’t go.* [FG4]

*Yes, I definitely have to arrange everything.* [FG2]

*I want to make sure that I am not going to be someplace there is not a bathroom for eight hours.* [FG2]

In addition to planning daily activities around bowel symptoms after IPAA, multiple participants described the burden of having to change plans due to an unexpected increase in bowel symptoms. These adjustments could include altering previously established plans or altering activities, including diet, in response to symptoms:

*I find myself, if I’m having a bad day, having to back out of plans.* [FG1]

*Every couple of weeks… having a flare up where I’m altering what I wanted to do because I just don’t have the energy.* [FG1]

#### Diet

There was significant overlap between the diet theme and several other identified themes. Participants indicated that diet and nutrition played a key role in function and bowel symptoms after IPAA. However multiple participants expressed difficulty identifying a diet that would reliably help control their symptoms.

*I’ve never found a diet that would work.* [FG1]

Some participants related regret or concern regarding foods that were tolerable prior to IPAA but now are not included in a regular diet due to the possible impact on bowel symptoms. Others noted an increase in the variety of foods that they could eat after IPAA (as compared to when they had medically refractory UC).

*My biggest regret is foods that I could eat before that I can’t now.* [FG1]

*I can tell that the more I eat, the more bowel movements I have.* [FG2]

## Discussion

In this study, we identified several important themes related to symptoms patients experience after an IPAA surgery and identified how these symptoms might relate to larger assessments of QoL and daily activities. The need to better understand and assess patients’ experiences after proctocolectomy with IPAA for UC has been demonstrated in recent years,^1,2^ and a significant disconnect remains with respect to assessments of pouch function (and dysfunction) between patients and clinicians.^10^ Additionally, a lack of consistency and reproducibility in patient-centered assessments after IPAA has created inconsistencies in methods and clinical practice that threaten rigor of research in this domain.

There is a critical need to better define the symptoms that are most common and impactful to patients after IPAA surgery. In a prior study comparing the perception of pouch dysfunction and resultant impact on QoL between patients and clinicians, the answers of participating clinicians (including surgeons and gastroenterologists) were no better than random probability in identifying the symptoms that were most important to patients after IPAA.^10^ Compared to patient responses, clinicians overestimated the importance of frequency of bowel movements and uncontrolled loss of stool, while underestimating the importance of urgency and incomplete evacuation.^10^ In our analysis, incontinence was a predominant symptom reported by patients and was linked to other domains including sleep and a sense of worry. Given the association of increased risk of nocturnal incontinence as the time from pouch surgery increases,^11^ particularly among patients greater than 65,^12^ a continued evaluation of the impact of these symptoms among younger patients may be informative.

In an assessment of over 1000 patients from Denmark, urgency was identified as having the greatest impact on QoL after IPAA surgery.^13^ Interestingly, among patients in our focus groups, although urgency played a key role in QoL, there was often a noted improvement in urgency after IPAA versus prior to colectomy. Patients undergoing IPAA for UC have a relatively unique opportunity to compare symptoms and life experiences with an IPAA (or even with a temporary ostomy) to those with UC prior to colectomy. In many of our discussions, participants compared their current symptoms or lived experience after IPAA surgery to those with medically refractory UC. Participants noted an improvement in many areas with direct effects on QoL after IPAA as related to the experience of having medically refractory UC, including bowel frequency, urgency, and pain. Additionally, many participants relayed their desire to avoid returning to an ostomy (and thus to preserve pouch function). Patients’ lived experiences pre- and post-IPAA could be explored in future work developing pouchitis- and IPAA-specific PROs.

The disconnect in how clinicians and patients view symptoms and QoL burden after IPAA for UC is likely further exacerbated in research and clinical practice by the use of broad PRO measures that lack specificity to pouchitis and other inflammatory conditions of the pouch.^1^ Although repurposing measures from other disease states, including CD, UC, and irritable bowel syndrome may address some aspects of the lived experience after IPAA, these nonspecific measures fail to fully capture the patients’ perception of their own pouch function, particularly as patients with IPAAs were not involved in validating these measures.^1^ Accordingly, dedicated assessments of the specific outcomes important to both patients and clinicians after IPAA will aid in the longitudinal assessment of patients with inflammatory conditions of the pouch and better assess response to therapy.

The US Food and Drug Administration (FDA) has documented the need to develop and/or validate PROs for the condition where they will be studied/utilized.^14^ Our observations demonstrate the breadth of experiences among patients after IPAA, including those with and without symptoms related to inflammatory conditions of the pouch. Involving patients in the development of future PROs specific to pouchitis or inflammatory conditions of the pouch will be critical to fully capturing the patient experience. The Ileoanal Pouch Syndrome Severity Index was developed with patient involvement at multiple phases and focuses on many of the areas identified in our focus group discussions.^6^ This measure assesses symptoms over the past 3 months, thus there are some questions on whether a shorter window of assessment will be necessary for use in clinical practice and research settings. However, it demonstrated predictive validity with respect to multiple measures during the development process, including the assessment of QoL after IPAA.

Our focus groups demonstrate the interconnectivity of bowel function and multiple QoL domains or themes. Most participants altered their daily activities in response to active bowel symptoms, and bowel symptoms significantly impacted participants’ overall QoL. In early studies of the Cleveland Clinic Global Quality of Life Scale,^4^ one of the most commonly utilized PRO measures after IPAA,^1^ there was notable overlap between bowel symptoms and QoL measures, including varying impacts of urgency and frequency on social activities, work activities, and diet restrictions over the first 8+ years after IPAA surgery.^4^ When viewed in conjunction with the symptoms and burdens described by participants in our study, it is evident there is a need for more granular assessments of these domains in future pouch-related PROs, particularly among patients with active symptoms of pouchitis or other inflammatory conditions of the pouch.

Dietary restrictions after IPAA have been long-recognized,^15^ and diet remains an area of significant interest for many patients, clinicians, and researchers evaluating individuals after IPAA for UC.^16–22^ However, heterogeneity among past study results limits our ability to draw evidence-based conclusions regarding the best dietary strategies post-IPAA.^20^ As evidenced in our discussions, many patients elect to alter their diets in response to bowel symptoms, which could result in nutritional deficiencies or dietary consumption patterns that fail to meet the current USDA nutritional recommended guidelines.^21^ Future research is needed to evaluate the feasibility of incorporating personalized nutrition assessments and subsequent recommendations into larger assessments of inflammatory conditions of the pouch.

Study strengths include representation from patients with a variety of pouch-related conditions. We intentionally recruited individuals with both normal pouch function and a variety of pouch-related disorders. Study limitations include the single-center design, as the patient experiences represented are from a single Multidisciplinary IBD Center and Multidisciplinary Pouch Clinic. Although our IBD Center serves a large catchment area across multiple states, the experiences described by the study participants may not be fully generalizable to the entire pouch population as they include only individuals who receive care from a select number of gastroenterologists and colorectal surgeons. Additionally, our population only included one Black patient and one patient of Hispanic ethnicity. More research understanding the lived experience after IPAA among patients with diverse racial and ethnic backgrounds is necessary.

As with all qualitative work, the demographic characteristics of the focus group leader, including sex, race, ethnicity, and occupation, could have potentially impacted participant responses and influenced our findings. As we expand this research we plan to utilize unaffiliated focus group leaders in future work. There is also the potential for selection bias among participants, given their willingness to participate in the focus group. We noted no variance in participation or transcripts based on the use of virtual or in-person focus groups. Although medical records were used to confirm diagnoses, we used patient self-report of symptoms and disease state at the time of focus group interviews rather than objective evaluation or validation of active inflammation. Similarly, we did not record a detailed history of the patients’ medical care related to their IPAA surgery or subsequent pouch-related care. Despite participants reporting improvement with selected therapies for inflammatory conditions of the pouch, we did not record therapies for individual patients to stratify these responses by individual therapy utilized.

## Conclusion

We utilized a series of focus group interviews to improve our understanding of the patient experience after IPAA and to determine the pouch-related symptoms that matter most to patients and associated effects on QoL. Our findings indicate that bowel symptoms are robustly associated with QoL issues, including daily activity planning and symptom burden. We believe that development of PROs specific to the symptoms of pouchitis and pouch-related disorders would better define the experience after IPAA surgery and thus improve care and assessment of outcomes for patients post-IPAA for UC.

## Data Availability

Requests regarding raw data can be submitted to the corresponding author.
